# Wave energy budget analysis in the Earth’s radiation belts uncovers a missing energy

**DOI:** 10.1038/ncomms8143

**Published:** 2015-05-15

**Authors:** A.V. Artemyev, O.V. Agapitov, D. Mourenas, V.V. Krasnoselskikh, F.S. Mozer

**Affiliations:** 1 LPC2E/CNRS, 3A, Avenue de la Recherche Scientifique, 45071 Orleans Cedex 2, France; 2 Space Sciences Laboratory, University of California, 7 Gauss Way, Berkeley, California 94720, USA; 3 CEA, DAM, DIF, F-91297 Arpajon Cedex, France

## Abstract

Whistler-mode emissions are important electromagnetic waves pervasive in the Earth’s magnetosphere, where they continuously remove or energize electrons trapped by the geomagnetic field, controlling radiation hazards to satellites and astronauts and the upper-atmosphere ionization or chemical composition. Here, we report an analysis of 10-year Cluster data, statistically evaluating the full wave energy budget in the Earth’s magnetosphere, revealing that a significant fraction of the energy corresponds to hitherto generally neglected very oblique waves. Such waves, with 10 times smaller magnetic power than parallel waves, typically have similar total energy. Moreover, they carry up to 80% of the wave energy involved in wave–particle resonant interactions. It implies that electron heating and precipitation into the atmosphere may have been significantly under/over-valued in past studies considering only conventional quasi-parallel waves. Very oblique waves may turn out to be a crucial agent of energy redistribution in the Earth’s radiation belts, controlled by solar activity.

## Abstract (web summary)

Whistler-mode waves regulate trapped electrons in the magnetosphere, but an accurate determination of their energy budget has remained elusive. This study presents a full analysis of their magnetic and electric field contributions and finds that a large amount of energy is stored in oblique waves.

## Graphical abstract

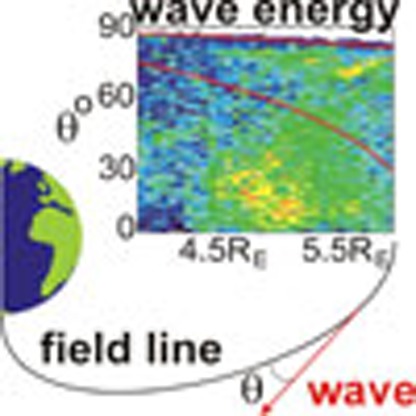

Since whistler-mode waves regulate fluxes of trapped electrons^1^,^2^ and their precipitation rate^3^,^4^,^5^ in the upper atmosphere^6^,^7^,^8^, accurately determining the wave energy budget in the outer radiation belt has lately become an outstanding challenge for the scientific community^9^. Owing to the sparse wave data obtained by early satellites, their poor coverage of high latitudes and mainly one-component field measurements, and as linear theory was showing much higher parallel wave growth, scientists have commonly relied on the assumption that chorus waves were mainly field-aligned, that is, their propagation was weakly oblique with respect to the geomagnetic field^10^,^11^. Moreover, crucial theoretical works^12^,^13^ in this area have demonstrated early on that the most important wave field component determining the wave–particle coupling efficiency was generally the magnetic field one, at least over a reasonably large range of wave obliquity. As a result, previous wave statistics focused on the sole magnetic field component of the full wave energy—showing indeed a clear prevalence of parallel waves in the equatorial region sampled by most satellites^14^,^15^,^16^,^17^.

No global study on the basis of satellite measurements since then has led to a real revision of this conventional picture. Although some studies^16^,^17^,^18^,^19^ and ray-tracing simulations^20^ have recently hinted at both the possible presence and potential importance of very oblique whistler-mode waves, they failed to grasp the full extent of the implications, owing either to their continuing focus on statistics of the sole magnetic field component or to their use of statistical averages over such wide ranges of geomagnetic conditions that the effects of oblique waves have become blurred. Here, we study the full wave energy distribution of whistler waves, including both magnetic and electric field components. Our work suggests that the unexpected presence of a very large electrostatic energy, hitherto missing in past statistics of wave intensity and stored in very oblique waves, may profoundly change the current understanding of both the actual wave generation mechanisms and the processes of wave-induced electron scattering, acceleration and loss in the magnetosphere.

## Results

### Statistics of wave energy

**Figure 1:**
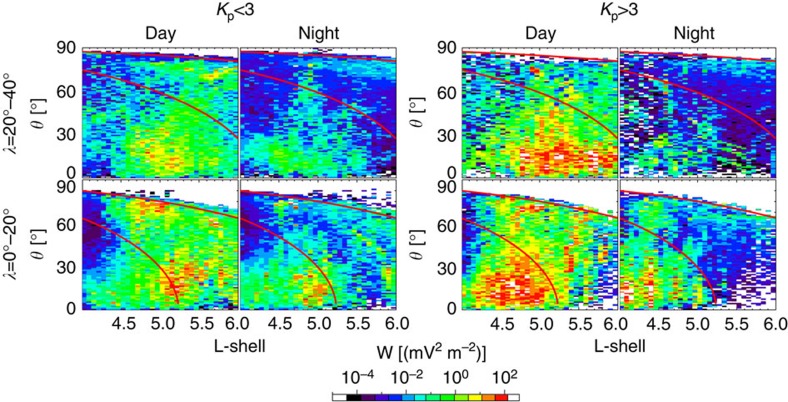
Distribution of the energy of whistler waves in the Earth radiation belts. The distribution of the density of whistler wave energy *W* (in mV^2^ m^−2^) is displayed in the (L,*θ*) space. Data are shown for two ranges of magnetic latitude (the near-equator region with |*λ*|∈[0°,20°] and the high-latitude region with |*λ*|∈[20°,40°]), for day and night sectors, and for low (*K*_*p*_<3) and high (*K*_*p*_>3) geomagnetic activity. Red curves show the position of Gendrin *θ*_g_ and resonance cone *θ*_r_ angles (where cos*θ*_g_≈2*ω*/Ω_*c*_, cos*θ*_r_≈*ω*/Ω_*c*_ and Ω_*c*_ is the local electron gyrofrequency). Both angles are calculated with the mean frequency of spacecraft observations, making use of precise plasma density measurements from IMAGE^39^. In the present figure, the wave refractive index has been limited to <100 in agreement with rough but conservative upper bounds due to Landau damping by average levels of hot electrons^19^. Three frequency channels have been taken into account: 2,244.9, 2,828.4 and 3,563.6 Hz, covering almost the full range from 2 to 4 kHz. Each channel is used in the corresponding L-shell range to measure only waves in whistler-mode frequency range.

To compare the impact of oblique and parallel waves in the formation and evolution of keV to MeV electron fluxes in the inner magnetosphere, a reasonable approach consists in first estimating the energy density of both wave populations. Such a global survey is presented in Fig. 1. Here, we make use of 10 years of wave measurements performed by Cluster satellites^16^ to evaluate the wave energy distribution throughout much of the Earth’s inner magnetosphere as a function of wave obliquity and L-shell (the equatorial distance to the centre of the Earth normalized to Earth’s radius). The energy density *W* of whistler-mode waves is determined by wave electric **E** and magnetic **B** field vectors through a complex relationship involving the tensor of absolute permittivity (see equation (1) in Methods section). Using the cold plasma dispersion relation for whistlers, *W* depends only on wave characteristics such as magnetic amplitude *B*, frequency *ω*, wave-normal angle *θ* (which defines the wave obliquity with respect to the geomagnetic field) and refractive index *N*=*kc*/*ω* (with *k* is the wave vector and *c* is the velocity of light).

Figure 1 with two-dimensional maps of wave energy *W* clearly shows that the proportion of very oblique waves, propagating near the resonance cone angle (that is, near 90°), is generally similar to or even larger than the proportion of quasi-parallel waves for L=3 to 6 during moderate geomagnetic activity (defined by index *K*_*p*_<3) on the dayside. On the nightside or during more disturbed periods such that *K*_*p*_>3 (that is, geomagnetic storms or substorms), the amount of very oblique waves is sensibly reduced. The latter reduction stems probably from the presence of higher fluxes of hot (∼100 eV to 1 keV) plasmasheet electrons injected in the midnight region during disturbed periods^21^. Numerical ray-tracing simulations have shown that such hot electrons can damp very oblique waves propagating near their resonance cone^19^,^20^,^22^.

The present results therefore challenge the conventional assumption of predominantly quasi-parallel whistler-mode waves in the outer radiation belt. A big, missing slice of the wave energy appears to be stored in very oblique waves—which are mainly made up of electrostatic energy^23^. Although most oblique waves are observed away from the equator, significant amounts moreover exist close to it. It strongly suggests that the widely accepted theory of parallel wave generation near the equator by an unstable electron population exhibiting a temperature anisotropy^11^,^24^,^25^ might need to be supplemented with some new mechanism allowing the direct generation of very oblique waves there. This could require the presence of additional energetic electron populations differing subtly from the commonly assumed ones.

**Figure 2:**
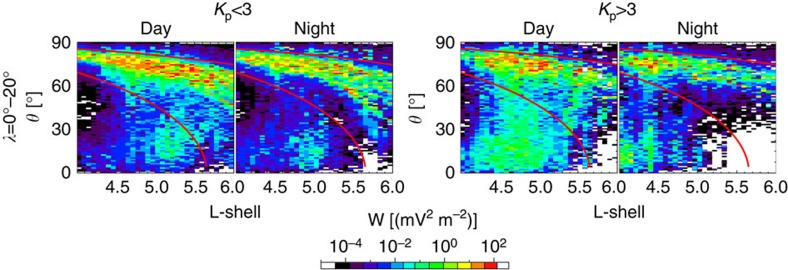
Distribution of the energy of resonant whistler waves in the radiation belts. The distribution of the wave energy density Θ^2^ of resonant waves (in mV^2^ m^−2^) is displayed in the (L,*θ*) space. The most effective resonant wave–particle interaction corresponds to a condition tan *α* tan *θ*≈1 (where *α* is the particle pitch angle) for electron energy <2 MeV (ref. 31). This condition has been used to plot Θ^2^ in this figure. Data are shown for one range of magnetic latitude |*λ*|∈[0°,20°], for day and night sectors, and for low (*K*_*p*_<3) and high (*K*_*p*_>3) geomagnetic activity. Red curves show the position of Gendrin *θ*_g_ and resonance cone *θ*_r_ angles. Both angles are calculated with the mean frequency of spacecraft observations, making use of precise plasma density measurements from IMAGE^39^. In the present figure, the wave refractive index has been limited to <100 in agreement with rough but conservative upper bounds due to Landau damping by average levels of hot electrons^19^. Three frequency channels have been taken into account: 2,244.9, 2,828.4 and 3,563.6 Hz, covering almost the full range from 2 to 4 kHz. Each channel is used in the corresponding L-shell range to measure only waves in whistler-mode frequency range.

What are the consequences of the large energy of oblique waves on the dynamics of energetic particles? As their name suggests, wave–particle resonant interactions are controlled not only by wave intensity, but also by the actual efficiency of the resonant interactions. Waves must be in resonance with particles, implying that a certain relationship must be fulfilled between particle energy, pitch angle, wave frequency and obliquity (see equation (2) in Methods section). As a result, only a small portion of the total wave energy density actually corresponds to resonant waves^12^. The wave–particle coupling efficiency *Φ*, which depends also on cyclotron or Landau resonance harmonic number and on wave field components, provides the exact portion of wave energy interacting resonantly with particles^13^,^26^, finally yielding the resonant wave energy Θ^2^=*B*^2^*Φ*^2^/*N*^2^. Figure 2 shows the wave energy density Θ^2^ of resonant waves plotted in the same manner as the wave energy density previously, using measured wave field components from Cluster. Figure 2 demonstrates that the resonant wave energy density at high *θ*-values (between the Gendrin and resonance cone angles) is 5–10 times larger than for parallel waves throughout the region L=3–6. Hence, very oblique waves are expected to play a crucial role in the scattering of electrons in this region of space.

### Electron lifetimes during geomagnetic storms

**Figure 3:**
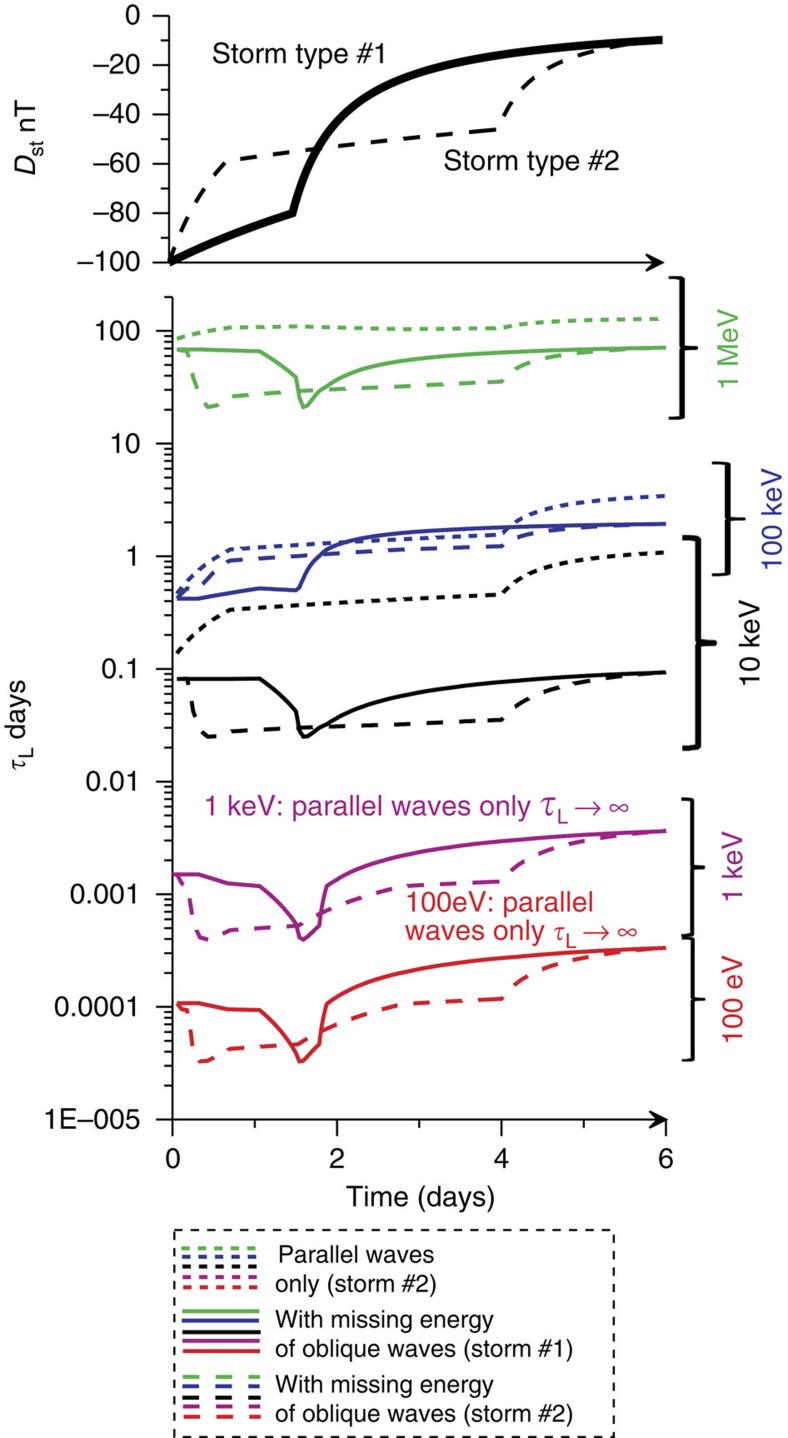
Variation of electron lifetimes in the course of geomagnetic storms. Two different temporal profiles of the *D*_st_ index are shown in the top panel, corresponding to two different types of storms. Bottom panels show the corresponding variation of electron lifetimes *τ*_L_ calculated for L∼5 at different energies (each colour corresponding to one energy). Dotted lines correspond to *τ*_L_ for parallel chorus whistler-mode waves (*τ*_L_ is infinite for 100 eV and 1 keV in this case) in the case of storm type #2. Solid (storm type #1) and dashed (storm type #2) lines show *τ*_L_ when using a realistic distribution of wave-normal angle. Five energies are shown: 100 eV, 1 keV, 10 keV, 100 keV and 1 MeV (for *E*≤ (>)1 keV, lifetimes are numerically calculated for electrons of equatorial pitch angle <60°(85°)).

The remarkable effectiveness of the resonant interaction of very oblique waves with keV to MeV electrons can modify particle scattering and energization processes substantially in the radiation belts as compared with conventional theoretical estimates obtained for quasi-parallel waves alone. This effect should be most pronounced during moderately disturbed periods where oblique waves are more ubiquitous. To estimate the effects of oblique waves on resonant electron scattering during the course of a geomagnetic storm, we use here parameterizations of lower-band chorus wave magnetic intensity and *θ* distributions as functions of *D*_st_ devised on the basis of the same wave data set^18^,^27^. The disturbance storm time *D*_st_ index is widely used to study the magnitude and internal variability of geomagnetic storms^28^,^29^. Two typical profiles *D*_st_(*t*) are considered (see top panel in Fig. 3), corresponding to storm types #1 and #2 (refs 28, 30). The storm type #1 has a relatively long (∼1.5 day) early recovery phase between *D*_st_=−100 and −75 nT followed by a rapid increase of *D*_st_ back to −10 nT, while the second type has a much shorter early recovery phase followed by a much more prolonged stay (∼3 days) around −50 nT.

The evolution of the lifetime *τ*_L_ of energetic electrons during the course of these two storms has been calculated numerically for various energies ranging from 100 eV to 1 MeV. Figure 3 first demonstrates the important variations of *τ*_L_ with *D*_st_. Such strong variations can be explained by the combination of two main effects: (1) lifetimes increase when wave intensity decreases (both with and without oblique waves)^10^,^31^ and (2) the wave–particle coupling *Φ* is significantly stronger for very oblique waves than for quasi-parallel waves over a very wide energy range (see Supplementary Fig. 2), leading to a reduction of lifetimes as the amount of very oblique waves increases during not-too-disturbed geomagnetic conditions^18^,^19^,^27^. The number of contributing resonances can moreover increase up to 10-fold for very oblique waves (see discussion of Supplementary Fig. 2 in Methods section).

When considering a realistic wave-normal angle distribution, the first clear consequence of the additional presence of very oblique waves is a general reduction of lifetimes during the storm. Most remarkably, however, such a reduction is much less significant during the early recovery period corresponding to *D*_st_<−75 nT. The latter range actually corresponds to high parallel wave amplitudes. Very oblique waves are then almost absent, probably due to their quick damping by intense injections of hot electrons during the main phase of strong storms. Thus, an extended storm phase such that *D*_st_<−75 nT, with intense waves and small losses, is particularly propitious for the strong energization of electrons. Later on, the competition between the opposite effects of a rapidly decreasing wave intensity and an increasing amount of oblique waves as *D*_st_ increases, results in a local minimum of *τ*_L_ near *D*_st_∼−60 nT during the early recovery phase. Finally, during nearly quiet periods with *D*_st_∼−10 nT at the end of storms, electron losses to the atmosphere are significantly increased by oblique waves, especially at very low energy. The remarkable difference between *τ*_L_ calculated for parallel waves alone and with a realistic *θ*-distribution reaches indeed one order of magnitude for 10 keV electrons, while at lower energies (≤1 keV), only very oblique waves are still able to resonantly scatter electrons towards the loss-cone.

## Discussion

Such results definitely show that a precise knowledge of the actual distribution of wave energy as a function of propagation angle *θ* is a key factor for accurately modelling the evolution of relativistic as well as low-energy electron fluxes under the influence of resonant wave–particle interactions. As noted above, this *θ* distribution is tightly controlled by the density and temperature of hot electrons. The large energy stored in very oblique waves can be readily tapped by sufficiently hot electrons newly injected from the outer magnetosphere and lead to their further heating via Landau damping. It, therefore, represents an accelerating factor of change for this important population^25^ of particles. More generally, the intrinsic variability of hot electron injections with geomagnetic activity^21^ probably explains the observed variation of wave obliquity^18^,^27^. As the latter is able to fine-tune the precipitation of very low energy (especially 0.1 to 10 keV) electrons, the presence of a large amount of very oblique waves could have unexpected and major consequences on the ionospheric conductivity and on the nightside upper atmosphere ionization level at various altitudes, potentially affecting the whole magnetosphere–ionosphere coupling^7^,^8^,^32^.

Beside determining electron precipitations into the atmosphere, whistler-mode waves are also responsible for the rapid energization of ∼10 keV to 1 MeV electrons to multi-MeVs in the radiation belts during geomagnetic storms^3^,^5^,^33^. To first order, the effective energization depends mainly on the dimensionless product *D*_EE_*τ*_L_ of the energy diffusion rate *D*_EE_ and lifetime, because a longer *τ*_L_ leaves more time for electron acceleration to proceed^18^,^27^,^34^. Moreover, the important dependence of the energy diffusion rate on the wave magnetic intensity (strongly increasing with −*D*_st_) is almost fully compensated in this factor *D*_EE_*τ*_L_ by the inverse dependence of the lifetime on the wave intensity. Since *D*_EE_ varies also much more weakly with wave obliquity than *τ*_L_^35^, it is the important variation of the lifetime with wave obliquity that should mainly determine the variation of the effective energization level of electrons. Thus, the comparison of lifetimes calculated with and without very oblique waves in Fig. 3 directly demonstrates the often dramatic change in energization level between these two cases.

Furthermore, the results displayed in Fig. 3 suggest that two storms with the same maximal strength but with different temporal profiles may lead to different effects on energetic electron fluxes, because of the different lifetime reductions dictated by the varying amount of very oblique waves. A storm (close to type #1) with a prolonged early recovery phase at *D*_st_<−75 nT followed by a quick return to *D*_st_>−20 nT should take advantage of high parallel wave intensity and weak overall losses to strongly energize electrons. Conversely, another storm (close to type #2) with a shorter initial period at *D*_st_<−75 nT followed by a much slower recovery back to *D*_st_>−20 nT should generally involve much stronger electron losses induced by larger amounts of very oblique waves during the early recovery phase (up to 1.5 day in Fig. 3), associated with a smaller magnetic wave intensity—efficiently reducing electron energization during that period. Later, significant losses to the atmosphere combined with modest wave intensity should nearly prevent any substantial acceleration. This could help to answer one outstanding question in radiation belt physics—why some geomagnetic storms correspond to global electron energization, while other storms with the same magnitude of *D*_st_ variation do not^29^.

Excluding oblique waves from consideration would actually make the dimensionless energization factor *D*_EE_*τ*_L_ almost constant and independent of the *D*_st_ time profile, as it does not depend on the bounce-averaged wave intensity. Only the consideration of an additional dimension of the system, corresponding to wave obliquity, gives a chance to obtain a significant variation of particle acceleration efficiency with *D*_st_ and, as a result, immediately produces a difference in particle acceleration for different *D*_st_(*t*) profiles. This effect allows to separate precipitation-dominated storms with a fast early recovery further slowing down, from acceleration-dominated storms with a slow initial recovery later on speeding up.

The surprisingly high level of very oblique wave energy discovered in Figs 1 and 2 and the strong concomitant increase of the wave–particle coupling strength have revealed that the wave obliquity, regulated by low-energy electrons injected from the plasmasheet, represents a new and important lever governing the variations of energetic electron fluxes. It indicates one possible answer to the problem of often-noted discrepancies between modern radiation belt models and observations^9^,^36^. The consideration of only parallel waves mostly restricts the space of wave model parameters to a single parameter—the wave amplitude. However, the distributions of wave amplitudes with geomagnetic activity and spatial location are well documented^10^,^16^ and included in modern codes. In this study, we have clearly shown that there exists at least one additional model parameter—wave obliquity, which can control both the energization of electrons, their precipitation into the atmosphere and even the energy range of precipitated particles. The revelation of this hidden parameter and of the corresponding missing energy of very oblique waves should provide new opportunities to better understand and forecast the observed variations of energetic electron fluxes in the radiation belts as well as the global dynamics of the magnetosphere–ionosphere coupling.

## Methods

### Evaluation of the wave energy density

The energy density *W* of whistler-mode waves in the Earth’s magnetosphere is determined by wave electric field **E** and magnetic field **B** vectors through the relation:


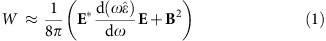


where E* is the conjugate vector to **E**, *ω* is the wave frequency and 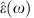 the tensor of absolute permittivity. Using the dispersion relation for electromagnetic whistler-mode waves in a cold magnetized plasma, electric field components can be further expressed as a function of **B**. One gets *W*=*B*^2^(1+*W*_*E*_)/8*π* where *W*_*E*_ depends only on the wave characteristics: its frequency *ω*, wave-normal angle *θ* (which defines the wave obliquity with respect to the geomagnetic field), and refractive index *N*=*kc*/*ω* (where *k* is the wave vector). *W* steeply increases with *N*, which is itself a rapidly growing function of *θ*. The refractive index *N* (as well as *θ*) can be determined either solely from full three-component wave magnetic field measurements on a given spacecraft, or else by complete wave magnetic and electric field measurements. The dominant contributions to the wave energy distribution can be further assessed on the basis of either method. However, wave electric field measurements on Cluster satellites are often noisy, at least much more than magnetic field measurements, limiting their use in practice to some case studies. Therefore, we have chosen to resort to the just-discussed method of determination of the full wave energy density on the basis of measurements of the wave magnetic components alone. Nevertheless, the accuracy and reliability of this method must first be demonstrated.

To this aim, we have compared the crucial *N* values obtained by the two methods in a series of Cluster observations of chorus waves displayed in Supplementary Fig. 1. The comparison of panels (a) and (b) shows clearly that wave activity can be identified not only in magnetic field fluctuations, but also in the concomitant variations of the electric field. Most of the wave-power is concentrated around ∼3 kHz—the ratio of wave frequency to electron equatorial gyrofrequency is *ω*/Ω_*c*0_∼0.35. Waves can be considered as very oblique when *θ* is comprised between the Gendrin angle *θ*_g_≈arccos(2*ω*/Ω_*c*0_) (which corresponds to wave propagation at a group velocity independent of frequency^37^) and the so-called resonance cone angle *θ*_r_∼arccos(2*ω*/Ω_*c*0_) (the upper bound on *θ* where the cold plasma refractive index *N* of whistler waves goes to infinity^38^). For events in Supplementary Fig. 1, we have *θ*_g_∼55–65° and *θ*_r_∼75–85°. Most observed whistler-mode waves are such that *θ*∈[60°,85°] and can, therefore, be classified as very oblique chorus waves. A substantial part of the wave energy density (see panel (d)) consists of such oblique waves. The large ratio *W*/*W*_*B*_≫1 shows that most of the energy density then comes from the wave electric field. More importantly, evaluations of the wave refractive index *N* from three-component measurements of the sole wave magnetic field yield values very similar to calculations making use of both magnetic and electric field components, attesting the reliability of the former method (compare panels (e) and (f)). The discrepancy does not exceed 25% on average, showing that this method can be safely used for evaluating the wave energy density.

However, only some part of the total wave energy density can actually interact resonantly with trapped electrons^12^. This resonant part is determined by the wave–particle coupling efficiency *Φ* (ref. 2) which depends on resonance harmonic number *n*, electron energy and pitch angle, as well as on the wave field components^13^,^26^. The resonance condition


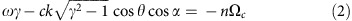


provides the necessary relation between particle energy (Lorentz factor *γ*), pitch angle *α* and wave obliquity *θ*. As a result, one gets a normalized estimate Θ^2^=*Φ*^2^*B*^2^/*N*^2^ of the resonant wave energy^13^.

### Evaluation of wave-particle coupling and diffusion rates

To demonstrate the peculiarities of electron resonant interaction with very oblique waves, additional numerical calculations of the wave–particle coupling efficiency *Φ* (averaged over latitude) have been performed as a function of wave propagation angle *θ* and geomagnetic activity index *D*_st_, for various electron energies ranging from 100 eV to 1 MeV. Here, as well as for Fig. 3, we use usual values of the mean frequency *ω*_m_/Ω_*c*0_∼0.35 and frequency width Δ*ω*/Ω_*c*0_∼0.2 of lower-band chorus waves^19^ and a ratio Ω_pe_/Ω_ce_=5 corresponding to L∼5. Supplementary Fig. 1 shows that during not-too-disturbed geomagnetic conditions (*D*_st_>−60 nT), wave–particle coupling *Φ* is clearly stronger for very oblique waves than for quasi-parallel waves over a wide energy range. For a given level of wave intensity, the available range of variation of the wave–particle coupling efficiency *Φ* at small equatorial pitch angles (near the loss-cone where particles are precipitated in the atmosphere) is so large that it could presumably explain any observed fluctuations of electron flux by fluctuations of the wave obliquity only and associated variations of electron scattering. In addition to the increase of *Φ* for a given resonance, the number of such contributing resonances can moreover increase 10-fold for oblique waves (see Supplementary Fig. 2).

The efficiency of charged particles resonant interaction with waves is determined by diffusion rates proportional to the weighting factor *Φ*_*n*_^2^=Θ^2^*g*_*θ*_(*θ*)*g*_*ω*_(*ω*) where *g*_*θ*_ and *g*_*ω*_ are normalized distributions of *θ* and wave frequency. To calculate the *g*_*θ*_ normalization, one should determine resonant *k* and *ω* for given particle pitch angle and energy. Then, an integration over *θ* must be performed. The upper limit of this integration is determined by the maximum value of the refractive index *N*_Max_. The latter is imposed by the presence of both thermal effects in the dispersion relation and Landau damping by 100–500 eV suprathermal electrons of oblique waves near the resonance cone angle^19^,^22^. Using typically observed parameters for the thermal and suprathermal electron population at L∼5, it has been shown that one could take *N*_Max_∼120 to 300 for lower-band chorus waves from low to high latitudes during periods of quiet to moderately disturbed geomagnetic activity, with *N*_Max_ varying as the inverse of the frequency *ω* and increasing with latitude^19^. It led us to use here (in Supplementary Fig. 2, and Fig. 3 in main text) a rough but realistic limit *N*_Max_∼min(300,36Ω_ce_/*ω*) corresponding to a predominant effect of Landau damping.

## Additional information

**How to cite this article:** Artemyev, A. V. *et al.* Wave energy budget analysis in the Earth’s radiation belts uncovers a missing energy. *Nat. Commun.* 6:7143 doi: 10.1038/ncomms8143 (2015).

## Supplementary Material

Supplementary Information Supplementary Figures 1-2
