# Publisher Correction: Wave energy budget analysis in the Earth’s radiation belts uncovers a missing energy

**DOI:** 10.1038/ncomms16197

**Published:** 2018-03-26

**Authors:** A. V. Artemyev, O. V. Agapitov, D. Mourenas, V. V. Krasnoselskikh, F. S. Mozer

Nature Communications 6: Article number: 7143; DOI: 10.1038/ncomms8143 (2015); Published: 05 15 2015; Updated: 03 26 2018

The original HTML version of this Article had an incorrect article number of 8143; it should have been 7143. This has now been corrected in the HTML; the PDF version of the Article was correct from the time of publication.

