# Supplementary material for: Wave energy budget analysis in the Earth’s radiation belts uncovers a missing energy
Source: Nat Commun. 2015 May 15;6:7143. doi: 10.1038/ncomms8143 (PMC4479018; doi:10.1038/ncomms8143)
Supplement: Supplementary Information — Supplementary Figures 1-2 [file ncomms8143-s1.pdf]

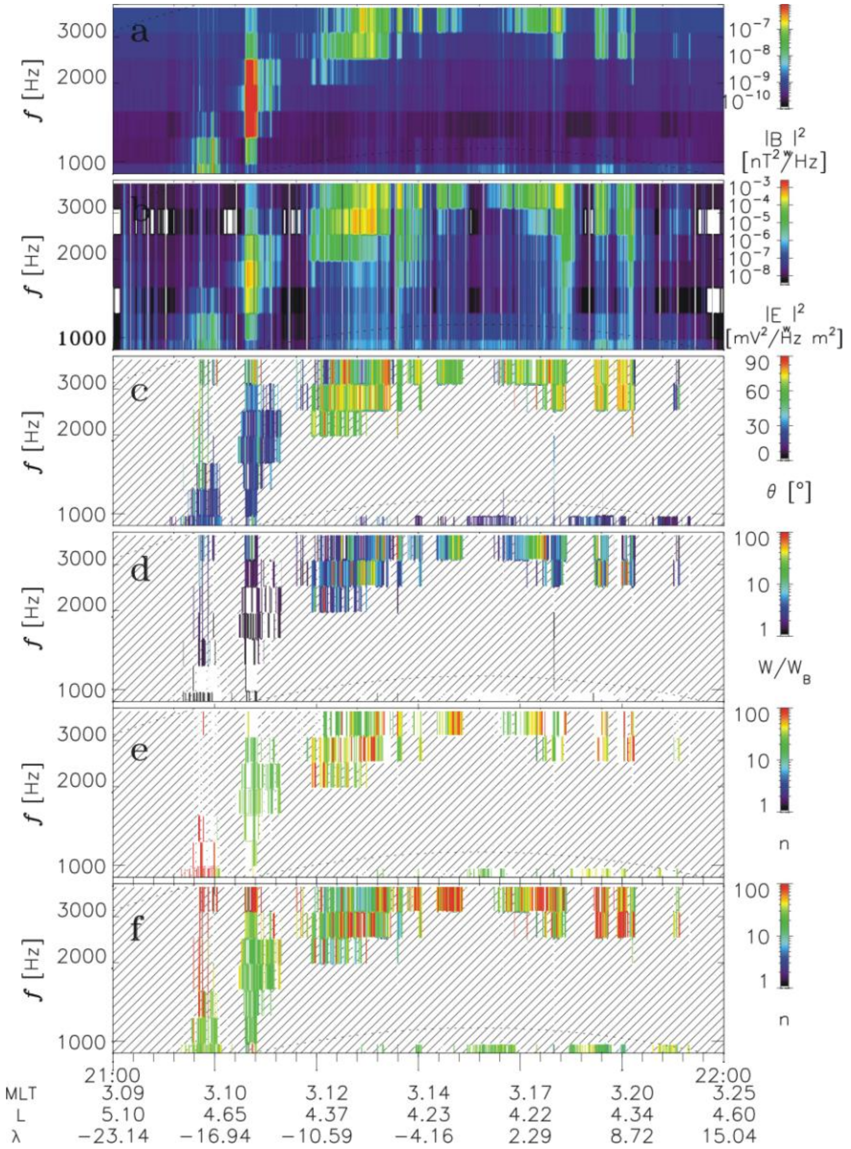

Supplementary Fig. 1

Example of Cluster observations of chorus whistler-mode wave activity at *L*~5. Top to bottom panels show: the distributions of power density of wave magnetic and electric fields, the distribution of wave-normal angle θ, the ratios of energies W/W_B calculated from the wave magnetic field components alone together with θ-estimation. The last two panels show the estimates of the refractive index *N* obtained from wave magnetic field measurements alone and directly from measurements of both the wave magnetic and electric field components. *N* often reaches ~100, but seldom goes much beyond that value at low latitudes. Landau damping by 100-500 eV electrons and thermal effects have been shown (Li et al. 2014) to lead to an upper-limit *N*<100 to *N*<300 from low to high latitudes during moderately disturbed geomagnetic conditions at *L*~5. It justifies using *N*≤100 as a rough but realistic and conservative upper-bound in the wave data analysis.

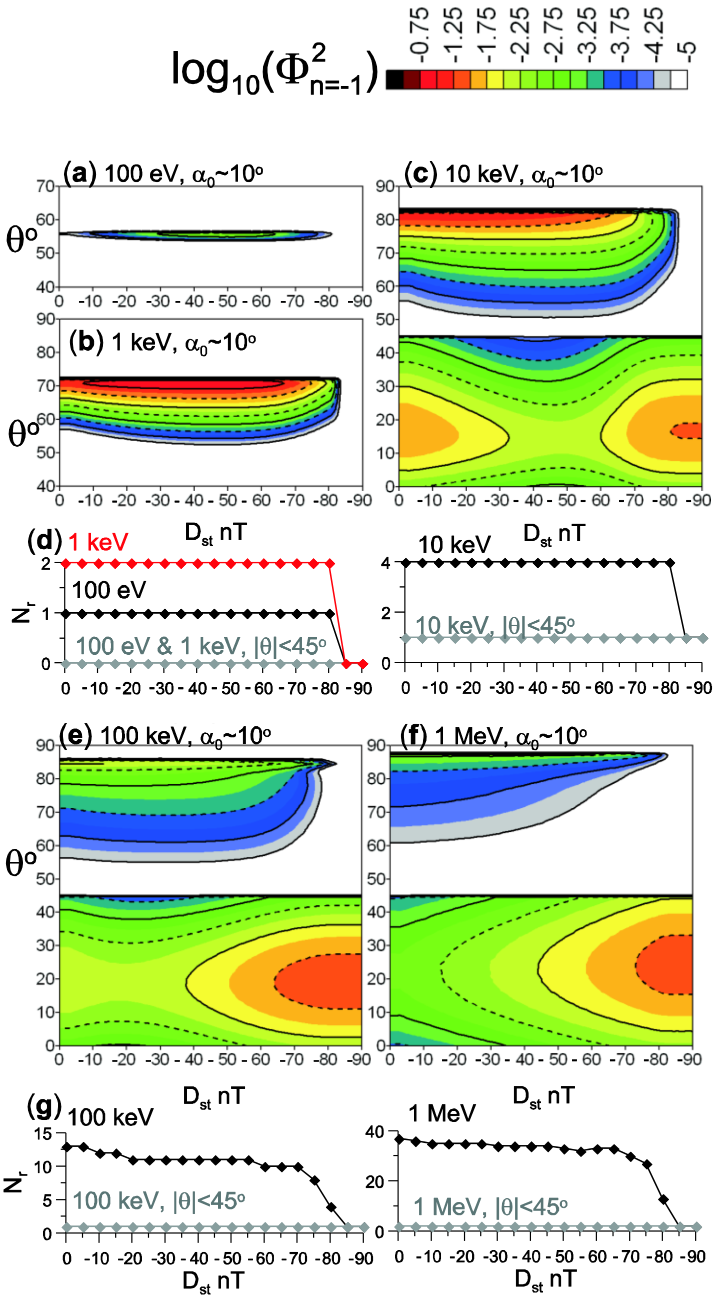

Supplementary Fig. 2

Wave-particle coupling efficiency Φ for the first-order cyclotron resonance. The panels (a, b, c, e, f) show the variations of the average wave-particle coupling efficiency Φ for the first-order cyclotron resonance in 2D maps as a function of wave obliquity θ and geomagnetic activity index D_{st} (this index characterizes the geomagnetic activity together with K_p; K_p<3 roughly corresponds to D_{st}>-50 nT) over equatorial pitch-angle α_0 ~5°-25°. Panels (d, g) show the corresponding number N_r of contributing cyclotron or Landau resonances for oblique vs. parallel waves (with individual contributions larger than 1% of the total diffusion rate). We consider L~5 and a rough but realistic latitude-varying upper-bound of N.
